# Representation and reporting of kidney disease in cerebrovascular disease: A systematic review of randomized controlled trials

**DOI:** 10.1371/journal.pone.0176145

**Published:** 2017-04-20

**Authors:** Ioannis Konstantinidis, Shanti Patel, Marianne Camargo, Achint Patel, Priti Poojary, Steven G. Coca, Girish N. Nadkarni

**Affiliations:** 1 Department of Medicine, Icahn School of Medicine at Mount Sinai, New York, New York, United States of America; 2 Division of Nephrology, Department of Medicine, Icahn School of Medicine at Mount Sinai, New York, New York, United States of America; 3 Department of Public Health, Icahn School of Medicine at Mount Sinai, New York, New York, United States of America; Istituto Di Ricerche Farmacologiche Mario Negri, ITALY

## Abstract

Patients with kidney disease (KD) are at increased risk for cerebrovascular disease (CVD) and CVD patients with KD have worse outcomes. We aimed to determine the representation of KD patients in major randomized controlled trials (RCTs) of CVD interventions. We searched MEDLINE for reports of major CVD trials published through February 9, 2017. We excluded trials that did not report mortality outcomes, enrolled fewer than 100 participants, or were subgroup, follow-up, or post-hoc analyses. Two independent reviewers performed study selection and data extraction. We included 135 RCTs randomizing 194,977 participants. KD patients were excluded in 48 (35.6%) trials, but were less likely to be excluded from trials of class I/II recommended interventions (n = 7; 15.9%; p = 0.001) and more likely to be excluded in trials with registered protocols (45.5% vs. 22.4%; p = 0.007). Exclusion was lower in trials supported by academic or governmental grants compared to industry or combined funding (21.2% vs. 42.0% and 47.8%; p = 0.033 and 0.028, respectively). Among trials excluding KD patients, 24 (50.0%) used serum creatinine, 7 (14.6%) used estimated glomerular filtration rate or creatinine clearance, 7 (14.6%) used renal replacement therapy, and 19 (39.6%) used non-specific kidney-related criteria. Only 4 (3.0%) trials reported baseline renal function. No trials prespecified or reported subgroup analyses by baseline renal function. Although 19 (14.1%) trials reported the incidence of acute kidney injury, no trial examined adverse event rates according to renal function. In summary, more than one third of major CVD trials excluded patients with KD, primarily based on serum creatinine or non-specific criteria, and outcomes were not stratified by renal parameters. Therefore, purposeful efforts to increase inclusion of KD patients in CVD trials and evaluate the impact of renal function on efficacy and safety are needed to improve the quality of evidence for interventions in this vulnerable population.

## Introduction

Kidney disease (KD) is a major public health problem of growing incidence and prevalence [1]. Although cardiovascular disease is acknowledged as the leading cause of mortality in KD patients [2], the interplay between KD and cerebrovascular disease (CVD) is often overlooked. Nevertheless, the prevalence of CVD among patients with CKD is more than two-fold higher that of patients without CKD [3]. Both decreased estimated glomerular filtration rate (eGFR) and albuminuria are independent risk factors for CVD [4]. Even mild renal impairment is associated with surrogate cerebrovascular disease markers, confers higher future risk of ischemic and hemorrhagic cerebrovascular events, and predicts poor clinical outcomes after an index stroke [5]. In addition, increase in acute kidney injury in CVD hospitalizations might contribute to future KD burden in stroke survivors [6].

Shared factors, such as hypertension, diabetes mellitus and older age, and KD-specific pathophysiologic mechanisms, including higher risk for atrial fibrillation, platelet and endothelial dysfunction, vascular calcification, and the malnutrition-inflammation-atherosclerosis syndrome, likely mediate the increased risk for CVD in the KD population [7]. Moreover, KD is associated with worse outcomes in functional impairment and in-hospital mortality among stroke patients. Finally, the efficacy and safety of interventions in acute stroke management and secondary stroke prevention are likely influenced by the kidney disease milieu.

However, there is a paucity of quality evidence about CVD therapies in KD patients as well as the interaction of kidney function with various interventions. We aimed to assess the representation of KD patients, reasons for exclusion, and reporting and analysis of baseline kidney function in major RCTs of CVD interventions.

## Materials and methods

The protocol of this systematic review was made *a priori* based on the guidelines of the Cochrane Handbook for Systematic Reviews of Interventions 5.1.0 and is reported according to the PRISMA statement (S1 Protocol) [8, 9].

We used a list of major medical journals based on journals’ impact factor over a sixteen-year period and examined trials regardless of whether they tested interventions that are class I or class II recommendations in current practice guidelines. The reason for this decision was twofold: on the one hand, substantial lag time frequently exists between the publication of trials and the formulation of practice guidelines that incorporate new research findings; on the other hand, trials of class III interventions or of interventions currently lacking a recommendation both reflect and may potentially influence clinical practice. As a result, an assessment of the representation of KD patients in these CVD trials is warranted. We did not include small trials and trials that were published in abstract form only or in non-major medical journals as those are less likely to influence contemporary clinical management of CVD.

### Data sources

We performed a systematic search of the MEDLINE database through the PUBMED interface for the period from inception through July 18, 2015. The search strategy was conducted on July 18, 2015 and included articles indexed under both the Medical Subject Headings (Mesh) “Stroke” or “Intracranial Hemorrhage” and also the Publication Type “Randomized Controlled Trial.” The search was limited to studies of human patients of any age or sex without language restrictions. We restricted the search to medical journals that were listed as among the top ten by annual impact factor in at least one year from 1999 to 2014 in the fields of general internal medicine, neurology, cardiology and nephrology. These included 22 general internal medicine journals, 28 neurology journals, 34 cardiology journals, and 21 nephrology journals. We supplemented this search by reviewing additional references from identified articles, not limited to the above 105 major medical journals. A total of 1,572 citations were obtained. The search was updated on February 9, 2017 and identified 128 reports that were published in the interim.

### Study selection

Two review authors (SP, MC) independently reviewed the full manuscripts of identified citations in duplicate. Differences in opinions were settled by consensus and, if necessary, a third review author (GNN) resolved disagreements. Trials were included if they were randomized controlled trials of treatment of transient ischemic attack (TIA), ischemic stroke (IS), intracerebral hemorrhage (ICH), or subarachnoid hemorrhage (SAH), and had 100 or more participants randomized. Trials were excluded if they did not report mortality outcomes (either as an endpoint or in safety/adverse event analysis) or were subgroup (e.g. elderly only), follow-up, or post-hoc analyses of the original study. After application of the eligibility criteria, 135 trials were selected for data extraction.

### Data extraction

Two review authors (IK, SP) independently extracted relevant study characteristics for each study in duplicate using a standardized and piloted data extraction form. Disagreements were resolved by consensus and, if necessary, a third review author (GNN). Variables included: journal name, year of publication, study recruitment period, number of centers involved (single- vs multicenter), location (by continent of the first author; Europe, United States/Canada, or Asia/Australia/South America), funding source (academic or government grant, industry, both, or not specified), treatment intervention implemented for each randomization arm, whether these treatment interventions are listed as either class I or class II recommendations in the current American Heart Association/American Stroke Association guidelines on the management of acute ischemic stroke, aneurysmal subarachnoid hemorrhage, and spontaneous intracerebral hemorrhage and the secondary prevention of stroke in patients with stroke and transient ischemic attack [10–13], diagnostic category [TIA, IS, ICH, SAH, or stroke; alone or in any combination], whether the protocol was registered in a clinical trials registry, number of patients randomized, whether patients with KD were excluded, threshold of exclusion of KD patients based on laboratory measurements, such as serum creatinine, creatinine clearance, or eGFR, renal replacement therapy, or nonspecific qualitative term, whether presence of KD was an inclusion criterion, reported indices of baseline renal function for each randomization arm (i.e. mean or median creatinine, creatinine clearance or eGFR), reported proportion of participants with KD, hypertension, diabetes mellitus, smoking history, hyperlipidemia, atrial fibrillation, overweight/obesity, cardiac disease and prior CVD in each arm, number of subgroup analyses by any non-renal baseline characteristics, number of subgroup analyses by renal parameters (both planned *a priori* in the registered protocol or methods report and performed in the index report), presence of significant interaction between renal function and outcome, and rate of adverse effects (overall and according to renal function). For studies with multiple reports, the first published report of trial results was used. In the case of insufficient information in the index report, methods reports (without restriction to journal of publication) and registered protocols of the selected trials were reviewed to supplement and complete the data fields.

### Data analysis

Cerebrovascular disease trials were assigned to 6 periods (1983–1988, 1989–1994, 1995–2000, 2001–2006, 2007–2012, and 2013–2016) based on their year of publication. Journals that had published 5 or more included index reports were analyzed separately whereas journals with fewer than 5 index reports were analyzed collectively. Types of interventions were grouped into both specific categories as well as 4 broad categories (medications, procedures, medication and procedures, and other). Trials were grouped into 4 categories based on sample size: 100–499, 500–999, 1000–4999, or 5000 or more participants. Differences in exclusion of patients with renal disease by each trial characteristic were evaluated with Fisher’s exact test. Reference groups for each characteristic were chosen as the group with the lowest frequency of exclusion and/or clinical judgment. All analyses were performed with STATA Version 12, College Station, TX. *P* values of ≤0.05 were considered statistically significant.

## Results

### Exclusion and inclusion of kidney disease

**Fig 1:**
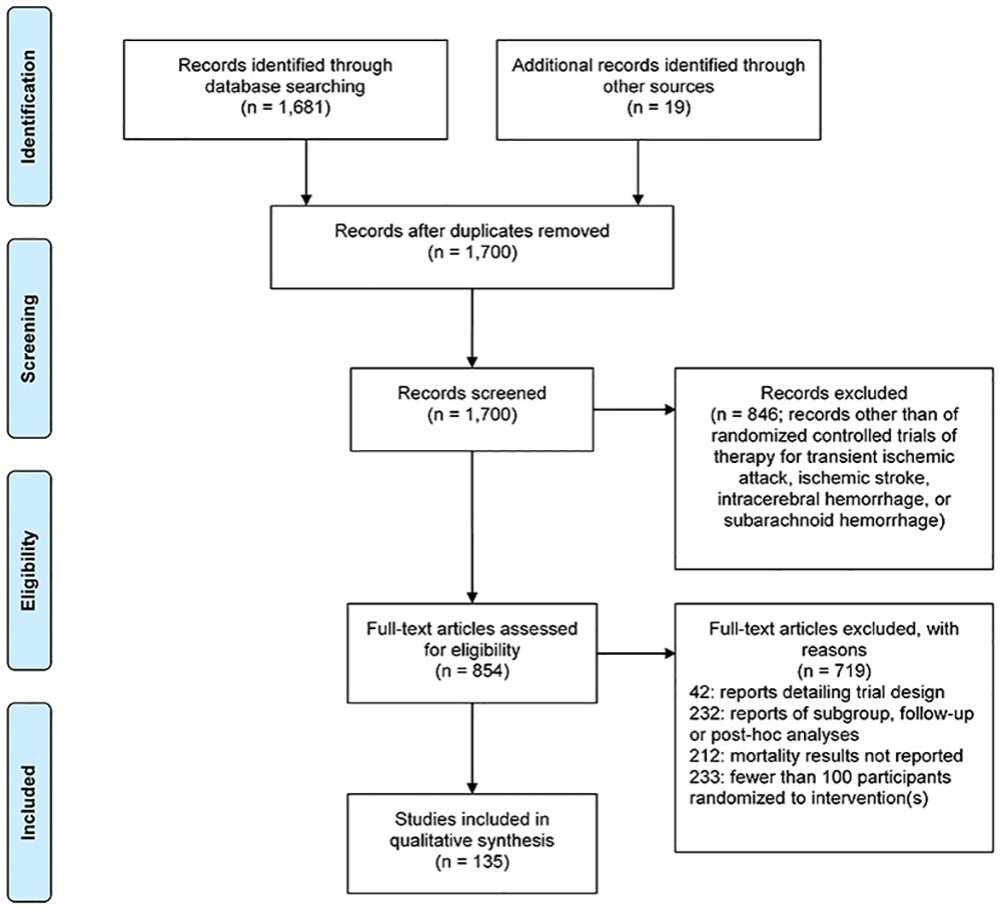
PRISMA flowchart regarding the search, screening, and selection of the identified articles.

**Fig 2:**
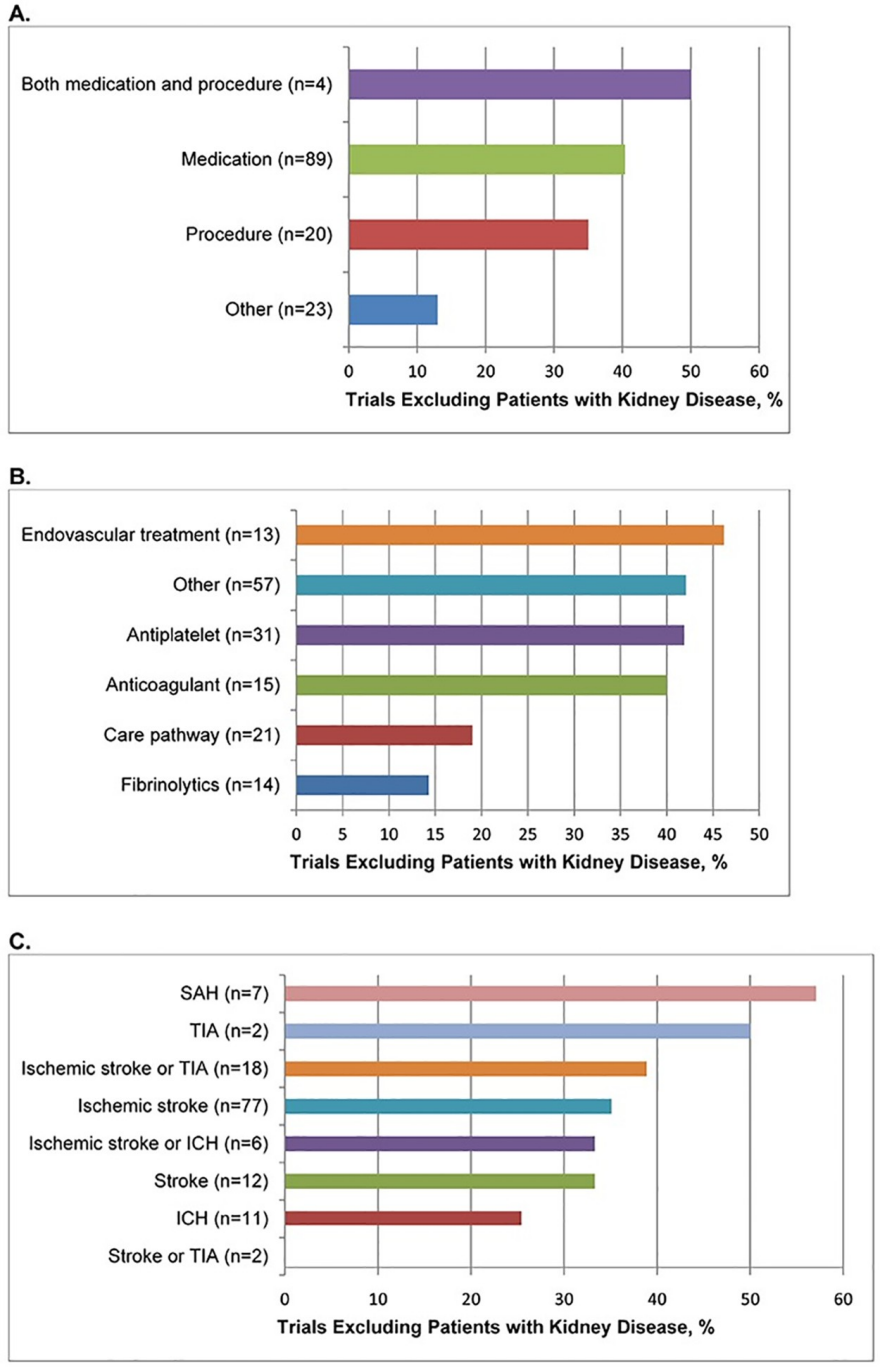
Bar chart of frequency of exclusion of patients with kidney disease in cerebrovascular trials by (A) Broad categories of treatment (B) Specific categories of treatment (C) Specific categories of diagnosis. Abbreviations: SAH (subarachnoid hemorrhage), TIA (transient ischemic attack), ICH (intracerebral hemorrhage).

A total of 135 trials randomizing 194,977 participants were included for analysis (Fig 1). Their characteristics are listed in Table 1. Overall, 48 (35.6%) trials excluded KD patients. Trials that tested interventions that have class I or II recommendations in current guidelines were less likely to exclude KD patients (p = 0.001). Exclusion was higher in trials that were registered (45.5% vs. 22.4%; p = 0.007), had moderately large sample size (50.0% for trials with 1,000–4,999 participants vs. 28.6% for trials with 100–499 participants; p = 0.035), and received industry or combined sponsorship compared to those that received solely academic/governmental grant funding (42.0% and 47.8% vs. 21.2%; p = 0.033 and 0.028, respectively). Exclusion was lower in trials that tested non-pharmacologic/non-procedural interventions compared to pharmacologic interventions (13.0% vs. 40.4%; p = 0.015). Frequency of exclusion did not differ by time period of publication, number of centers, location, and diagnostic categories. Frequencies of exclusion on the basis of treatment and diagnostic categories are presented in Fig 2.

**Table 1:** Characteristics of cerebrovascular trials.

	Trials	Number of Patients	Trials with Explicit Exclusion of Renal Disease Patients Based on Index Report, Methods Report of Registered Protocol, n	Percent	p value
**Overall**	135	194,977	48	35.6	NA
**Publication, year**					
• 1983–1988	4	2,617	0	0.0	Ref
• 1989–1994	3	493	1	33.3	0.429
• 1995–2000	15	8,905	2	13.3	1
• 2001–2006	34	52,765	10	29.4	0.556
• 2007–2012	42	76,220	17	40.5	0.281
• 2013–2016	37	53,977	18	48.6	0.118
**Class I/II recommendation**					
• Yes	44	74,249	7	15.9	Ref
• No	91	120,748	41	45.1	0.001
**Trial protocol registered**					
• Yes	77	133,474	35	45.5	Ref
• No	58	61,503	13	22.4	0.007
**Trial randomization size**					
• 100–499	63	16,915	18	28.6	Ref
• 500–999	27	18,120	8	29.6	1
• 1000–4999	38	82,902	19	50.0	0.035
• >5000	7	77,040	3	42.9	0.421
**Sites**					
• Single-center	12	2,769	3	25.0	Ref
• Multi-center	123	192,208	45	36.6	0.538
**Location**					
• Europe	70	91,431	20	28.6	Ref
• United States/Canada	43	73,395	20	46.5	0.069
• Asia/Australia/South America	22	30,151	8	36.4	0.596
**Funding source**					
• Academic grant/government	52	65,560	11	21.2	Ref
• Industry	50	93,418	21	42.0	0.033
• Both	23	26,314	11	47.8	0.028
• Not specified	10	9,685	5	50.0	0.108
**Journal**					
• Lancet	25	69,326	6	24.0	Ref
• Stroke	48	27,379	15	31.3	0.594
• NEJM	32	70,608	15	46.9	0.1
• Lancet Neurology	15	9,955	8	53.3	0.089
• JAMA	8	13,466	3	37.5	0.651
• Others (Archives of Internal Medicine, BMC Medicine, Circulation, Journal of Internal Medicine, Neurology)	7	4,243	1	14.3	0.006
**Diagnostic category**					
• Ischemic stroke	77	81,068	27	35.1	Ref
• ICH	11	11,279	3	27.3	0.743
• Ischemic stroke or ICH	6	6,874	2	33.3	1
• Ischemic stroke or TIA	18	64,585	7	38.9	0.789
• SAH	7	5,548	4	57.1	0.415
• Stroke	12	18,140	4	33.3	1
• Stroke or TIA	2	6,846	0	0.0	0.544
• TIA	2	637	1	50.0	1
**Therapeutic class—Broad**‡					
• Medication	89	159,539	36	40.4	Ref
• Procedure	20	10,083	7	35.0	0.801
• Both medication and procedure	4	1,674	2	50.0	1
• Other*	23	26,701	3	13.0	0.015
**Therapeutic class—Specific**‡					
• Antiplatelet	31	91,293	13	41.9	Ref
• Anticoagulant	15	30,565	6	40.0	1
• Fibrinolytics	14	10,211	2	14.3	0.094
• Care pathway	21	25,810	4	19.0	0.132
• Endovascular treatment	13	6,637	6	46.2	1
• Other†	57	63,729	24	42.1	1

*Other: educational material, care pathway, anesthesia plan.

^†^Other: ACEI, anesthesia plan, antibiotic, ARB, BB, benzothiazole derivative, CCB, defibrinogenating agent, educational material, endothelin receptor antagonist, EPO, factor replacement, free radical-trapping agent, GCSF, glycerol, hemicraniectomy, hemodilution, insertable cardiac monitoring, insulin, intercellular adhesion molecule-1 (ICAM-1) antibody, magnesium, nitroglycerin, NMDA receptor antagonist, platelet transfusion, peptide, PFO closure, remote ischemic preconditioning, sedative/hypnotic, serotonin agonist, statin, supplement, thiazolidinedione, transcranial laser treatment, US waves, uric acid, zinc chelator.

^‡^The sum of the number of trials based on the therapeutic class of the examined intervention is greater than the total number of trials, because several trials evaluated multiple interventions and thus were counted for each category. The sum of the percentages is greater than 100% for this reason.

Thresholds for exclusion of patients with KD are summarized in Table 2. Overall of 48 trials that excluded these patients, 24 (50.0%) used serum creatinine, 4 (8.3%) used eGFR, and 3 (6.3%) used creatinine clearance thresholds; 7 (14.6%) used renal replacement therapy of any form and 19 (39.6%) used nonspecific qualitative exclusion criteria (such as “significant renal impairment,” “nephropathy,” “renal dysfunction,” and “severe renal insufficiency”). No trial reported that patients with specific degrees of KD severity were recruited.

**Table 2:** Exclusion of kidney disease and reporting of renal function in cerebrovascular trials.

	All Trials n/N (%)	Intervention was class I/II recommendation, Trials n/N (%)
**Overall**	135 (100)	44/135 (32.6)
**Excluding renal disease based on the index report, methods report or registered protocol**	48/135 (35.6)	7/44 (15.9)
Threshold for exclusion*		
**Serum creatinine**	24/48 (50.0)	2/7 (28.6)
• Serum creatinine > 1.3–2.0 mg/dL (115–177 μmol/L)	6/48 (12.5)	0/7
• Serum creatinine ≥ 2.0–3.0 mg/dL (177–265 μmol/L)	12/48 (25.0)	1/7 (14.3)
• Serum creatinine ≥ 3.0 mg/dL (265 μmol/L)	6/48 (12.5)	1/7 (14.3)
**eGFR**	4/48 (8.3)	1/7 (14.3)
• eGFR ≤ 30 mL/min per 1.73 m2	3/48 (6.3)	0/7
• eGFR ≤ 60 mL/min per 1.73 m2	1/48 (2.1)	1/7 (14.3)
**CrCl**	3/48 (6.3)	1/7 (14.3)
• CrCl ≤ 30 ml/min	2/48 (4.1)	0/7
• CrCl ≤ 60 ml/min	1/48 (2.1)	1/7 (14.3)
**eGFR or CrCl**	7/48 (14.6)	2/7 (28.6)
• ≤ 30	5/48 (10.4)	0/7
• ≤ 60	2/48 (4.1)	2/7 (28.6)
**Renal replacement therapy**	7/48 (14.6)	2/7 (28.6)
**Nonspecific exclusion**	19/48 (39.6)	4/7 (57.1)
**Reporting baseline renal characteristics for each group in index report**		
• Baseline creatinine, eGFR or CrCl^†^	4/135 (3.0)	1/44 (2.3)
• Baseline creatinine	4/135 (3.0)	1/44 (2.3)
• Baseline eGFR	0/135	0/44
• Baseline CrCl	0/135	0/44
• Proportion of patients with renal disease	3/135 (2.2)	2/44 (4.5)
**Trials planning subgroup analyses by renal characteristics in registered protocol or methods report, if any**	0/85	0/28
**Trials reporting subgroup analyses in index report**		
• At least 1 subgroup analysis by baseline characteristics	69/135 (51.1)	19/44 (43.2)
• At least 1 subgroup analysis by nonrenal characteristics	69/135 (51.1)	19/44 (43.2)
• Average number of nonrenal subgroup analyses, mean (SD)	2.8 (4.0)	2.1 (3.6)
• At least 1 subgroup analysis by renal characteristics	0/135	0/44

*The sum of the number of trials for each category of exclusion criteria (serum creatinine, eGFR, CrCl, renal replacement therapy, and nonspecific exclusion) is greater than the total number of trials that excluded kidney disease patients, because several trials used multiple concurrent criteria for exclusion of kidney disease (e.g. serum creatinine and renal replacement therapy) and thus were counted for each category. The sum of the percentages is greater than 100% for this reason.

^†^The sum of the number of trials reporting for each baseline renal parameter (serum creatinine, eGFR, and CrCl) is greater than the total number of trials that reported baseline renal function, because several trials reported multiple renal parameters (e.g. serum creatinine and eGFR) and thus were counted for each category. The sum of the percentages is greater than 100% for this reason.

### Reporting on renal function and subgroup analyses

Only 4 (3.0%) trials reported baseline renal function, all of which reported baseline mean serum creatinine, and 3 (2.2%) trials reported the proportion of KD patients in each randomization arm (Table 2). No trials planned subgroup analyses *a priori* in the registered protocol or published methods report or reported subgroup analyses in the index report, whereas 57 (47.5%) trials reported subgroup analyses by one or more non-renal baseline characteristics. Although 19 (14.1%) trials reported the incidence of acute kidney injury during safety monitoring, no trial examined adverse event rates according to renal function.

**Fig 3:**
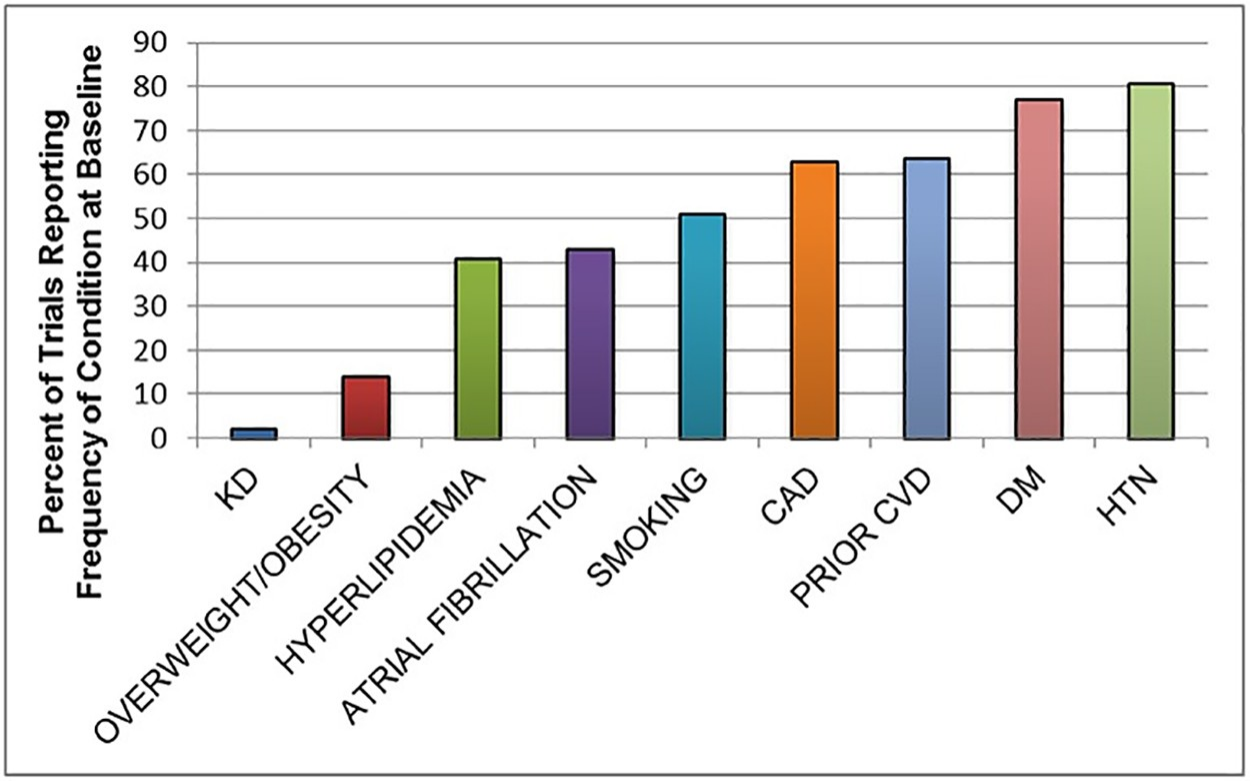
Bar chart of frequency of reporting of cerebrovascular disease risk factors in cerebrovascular trials. Abbreviations: KD (kidney disease), CVD (cerebrovascular disease), CAD (coronary artery disease), DM (diabetes mellitus), HTN (hypertension).

In contrast, trials reported the proportion of patients with other risk factors for CVD as follows: hypertension (n = 109; 80.7%), diabetes mellitus (n = 104; 77.0%), smoking history (n = 69; 51.1%), hyperlipidemia (n = 55; 40.7%), atrial fibrillation (n = 58; 43.0%), overweight/obesity (n = 19; 14.1%), cardiac disease (n = 85; 63%), and prior CVD (n = 86; 63.7%) (Fig 3).

### Subgroup analyses by class of recommendation

The characteristics of trials of class I/II treatment interventions are summarized in Table 3. A total of 44 trials of class I/II treatment interventions were identified randomizing 74,249 participants. Only 7 (15.9%) of trials excluded KD patients. Patients with KD were less likely to be excluded from European compared to North American trials and more likely to be excluded from moderately large trials. Frequency of exclusion did not differ by any other trial characteristic. Of the trials that excluded KD patients, serum creatinine and renal replacement therapy were each used in 2 trials, eGFR and creatinine clearance were each used in one trial, and nonspecific qualitative exclusion criteria were used in 4 trials. Baseline renal function was reported in a single trial, whereas 2 (4.5%) trials reported the proportion of patients with renal disease in each randomization arm. Subgroup analyses by one or more non-renal baseline characteristic were reported in 19 (43.2%) trials.

**Table 3:** Characteristics of cerebrovascular trials of class I/II treatment interventions.

	Trials	Number of Patients	Trials with Explicit Exclusion of Renal Disease Patients Based on Index Report, Methods Report of Registered Protocol, n	Percent	p value
**Overall**	44	74,249	7	15.9	NA
**Publication, y**					
• 1983–1988	2	1,109	0	0.0	Ref
•1989–1994	0	0	0	N/a	1
• 1995–2000	7	2,300	0	0.0	1
•2001–2006	11	17,435	1	9.1	1
•2007–2012	14	38,385	3	21.4	1
•2013–2016	10	15,020	3	30.0	1
**Trial protocol registered**					
• Yes	24	52,520	6	25.0	Ref
• No	20	21,729	1	5.0	0.106
**Trial randomization size**					
• 100–499	18	4,839	0	0.0	Ref
• 500–999	10	6,388	2	20.0	0.119
•1000–4999	13	31,070	4	30.8	0.028
• >5000	3	31,952	1	33.3	0.143
**Sites**					
• Single-center	4	667	0	0.0	Ref
• Multi-center	40	73,582	7	17.5	1
**Location**					
• United States/Canada	11	33,751	4	36.4	Ref
• Europe	22	22,555	0	0.0	0.008
• Asia/Australia	11	17,943	3	27.3	1
**Funding source**					
• Academic grant/government	25	30,891	3	12.0	Ref
• Industry	8	28,911	2	25.0	0.574
• Both	10	14,139	2	20.0	0.61
• Not specified	1	308	0	0.0	1
**Journal**					
• Lancet Neurology	6	3,040	1	16.7	Ref
• Lancet	7	13,569	0	0.0	0.462
• Stroke	11	9,509	0	0.0	0.353
• New England Journal of Medicine	15	41,168	6	40.0	0.613
• Others (Journal of American Medical Association, BMC Med, Journal of Internal Medicine)	5	6,963	0	0.0	1
**Diagnostic category**					
• Ischemic stroke	24	46,709	4	16.7	Ref
• ICH	6	9,026	2	33.3	0.571
• Ischemic stroke or ICH	2	942	0	0.0	1
• Ischemic stroke or TIA	5	7,342	1	20.0	1
• SAH	1	109	0	0.0	1
• Stroke	5	4,016	0	0.0	1
• Stroke or TIA	1	6,105	0	0.0	1
• TIA	0	0	0	N/a	1
**Therapeutic class—Broad**					
• Medication	25	52,155	5	20.0	Ref
• Procedure	4	1,207	1	25.0	1
• Both medication and procedure	0	0	0	N/a	1
• Other*	16	23,907	2	12.5	0.685
**Therapeutic class—Specific**^‡^					
• Antiplatelet	11	30,360	3	27.3	Ref
• Anticoagulant	8	25,627	2	25.0	1
• Fibrinolytics	8	9,196	1	12.5	0.603
• Care pathway	16	23,907	2	12.5	0.371
• Endovascular treatment	3	1,081	1	33.3	1
• Other^†^	6	12,888	1	16.7	1

*Other: care pathway

^†^Other: ACEI, ARB, BB, CCB, statin, US waves

^‡^The sum of the number of trials based on the therapeutic class of the examined intervention is greater than the total number of trials, because several trials evaluated multiple interventions and thus were counted for each category. The sum of the percentages is greater than 100% for this reason.

## Discussion

We systematically reviewed large, randomized controlled trials of interventions for cerebrovascular disease published in major medical journals through February 2017 to determine the representation and reporting of kidney disease. We found that more than 35% of major CVD trials excluded patients with KD. Further, most trials excluded patients based on serum creatinine or non-specific criteria rather than estimated glomerular filtration rate or creatinine clearance. Moreover, fewer than 5% of trials provided information on baseline renal function. No trial planned *a priori* or reported subgroup analyses in the index report by renal parameters. A minority of trials reported the incidence of acute kidney injury, however, none examined the rate of adverse events according to baseline renal function.

We previously reported that kidney disease patients were frequently excluded in major cardiovascular disease trials [14]. Although kidney disease is highly prevalent in the general population and among patients with cerebrovascular disease, KD patients are similarly underrepresented in CVD trials. This mirrors the historic underrepresentation of other subpopulations in clinical research, such as women, ethnic minorities, and the elderly [15–17], which prompted initiatives to increase their inclusion [18]. Researchers may exclude KD patients due to concerns that pathophysiological mechanisms of kidney disease (such as abnormal salt and water handling, platelet and endothelial dysfunction, and vascular calcification) may confer resistance to standard therapies, thereby decreasing the observed treatment effect in clinical trials and favoring the null hypothesis. On the other hand, patients with KD may be at increased risk for adverse effects related to routine therapies. For example, use of inhibitors of the renin-angiotensin-aldosterone system in the setting of kidney disease may have a higher risk associated with hyperkalemia and further renal impairment. Similarly, bleeding complications related to platelet dysfunction might be worrying to trialists of antiplatelet, anticoagulant, and fibrinolytic agents. Researchers of procedural interventions may exclude patients owing to the risk for worsening renal function following iodine administration during CT imaging and catheter angiography as well as the risk for nephrogenic systemic fibrosis related to the administration of gadolinium for MRI imaging. These concerns of decreased benefit and increased risk may help explain why trials with partial or exclusive industry sponsorship as well as those studies that examined medical and procedural therapies excluded KD patients more frequently than trials funded by academic or governmental grants and those that evaluated interventions, such as care pathways. Nevertheless, the reluctance to include patients with KD in cerebrovascular trials results in a missed opportunity to define the risk-benefit ratio of cerebrovascular therapies in a sizeable and vulnerable subpopulation, which is reflected by the relative insignificance of recommendations related to kidney disease in current guidelines on the management of ischemic stroke, intracerebral hemorrhage, and subarachnoid hemorrhage [10–13]. This is especially troublesome when one considers that the number of RCTs published in nephrology is low and characterized by poor quality, and therefore unlikely to influence cerebrovascular care in kidney disease patients. Consequently, beneficial therapies may be underutilized, whereas less effective or higher risk therapies may be overutilized in this population.

In addition to problems with exclusion of KD patients from CVD trials, the dearth of reporting on kidney disease as a baseline patient characteristic is alarming, because clinicians are unable to judge how relevant trials’ results are to a particular patient. Yet reporting on baseline renal function should be routine. First, it is warranted due to the high prevalence of kidney disease in CVD. Furthermore, renal function should be reported due to the potential impact that kidney disease may have on outcomes of CVD as well as the efficacy and safety of CVD therapies. Finally, necessary information for determining renal function (i.e. serum creatinine, age, sex, and race) is typically collected at enrollment and during follow-up. Nevertheless, kidney disease is reported in fewer than 5% of trials, unlike other baseline characteristics, such as hypertension, diabetes mellitus, coronary artery disease, prior CVD, smoking status, and atrial fibrillation, which are routinely described. At the same time, the reliance on serum creatinine is inappropriate given the availability of superior estimates of renal function, such as the glomerular filtration rate and creatinine clearance, which take into account age, gender, and race variations [19, 20].

Lastly, the absence of outcome and adverse event analyses according to baseline renal function is remarkable, especially given the possibility that concerns related to the risk-benefit of therapies in this population may be dissuading trialists from enrolling patients with kidney disease. Yet this review found that such analyses are neither planned *a priori* based on available protocols and methods publications, nor reported in the initial results publications. As a result, crucial information about the effect of renal function on the efficacy and safety of cerebrovascular interventions is lacking.

There are some limitations to this study. First, our search was limited to trials randomizing at least 100 patients. We cannot preclude the possibility that smaller trials may have been more likely to include patients with kidney disease. Nevertheless, our results were relatively consistent across sample size categories. Moreover, it is usually larger trials that are most likely to influence evidence-based medical practice. Second, this review included trials from major medical journals. As a result, some relevant studies may have been missed. However, our prespecified requirement for included journals to have been listed among the top ten by annual impact factor in at least one year over a sixteen-year period yielded a total of more than one hundred journals. As a result, most influential CVD trials were likely identified. Third, since the frequency of exclusion of kidney disease was greater in trials with registered protocols, we may have in fact underestimated the true rate of exclusion. Finally, the low rate of reporting on baseline renal function and lack of subgroup analyses stratified by renal function does not preclude the possibility that subsequent publications may have provided this information. Nevertheless, the lack of prespecified analyses as noted above means that subsequent reports would be susceptible to publication bias.

## Conclusions

In summary, trials of cerebrovascular therapies frequently exclude kidney disease patients, use suboptimal methods for quantifying renal function, and neither adequately report, nor analyze efficacy and safety outcomes by baseline renal function. As a result, the general standard of care for cerebrovascular disease should be cautiously extrapolated to patients with KD. Our findings underscore the need for greater inclusion of patients with kidney disease in future cerebrovascular trials. Government and industry sponsors should make such studies a priority and we recommend specific inclusion and exclusion criteria based on eGFR be used. Additionally, we urge trialists to report summary statistics for the presence of kidney disease and eGFR among participants, similarly to other baseline patient and laboratory characteristics. Finally, we encourage researchers to quantify the effect modification of outcomes and adverse effects related to therapies according to renal function. We believe that implementation of these recommendations will provide much-needed evidence to guide cerebrovascular care in this vulnerable and overlooked population.

## Supporting information

S1 Protocol Study protocol. (DOCX)

S1 Dataset Study dataset. (XLSX)

S1 PRISMA Checklist Prisma checklist. (DOC)
