# Supplementary material for: Representation and reporting of kidney disease in cerebrovascular disease: A systematic review of randomized controlled trials
Source: PLoS One. 2017 Apr 20;12(4):e0176145. doi: 10.1371/journal.pone.0176145 (PMC5398672; doi:10.1371/journal.pone.0176145)
Supplement: S1 Protocol — (DOCX) [file pone.0176145.s001.docx]

**Underrepresentation of Renal Disease in Randomized Controlled Trials of Cerebrovascular Disease: A Systematic Review**

**Objectives**: To evaluate representation of patients with renal disease in randomized controlled trials of drugs, procedures or devices for the prevention and treatment of cerebrovascular disease

**Team**: Ioannis Konstantinidis, Shanti Patel, Marianna Camargo, Achint Patel, Steven G Coca, Girish N Nadkarni

**Review Data**

Study Design: systematic review of randomized controlled trials

Key words: cerebrovascular disease;kidney disease;patient selection;systematic review;epidemiology

Funding and role: No funding specific for this review

The protocol of this systematic review was made *a priori* based on the guidelines of the Cochrane Handbook for Systematic Reviews of Interventions 5.1.0 (Higgins and Green, 2011) and is reported according to the PRISMA statement (Liberati et al., 2009).

**Methods**

**Criteria for considering studies for this review (Table 1)**

*Types of Studies*: Randomized controlled trials with 100 or more participants that reported mortality outcomes. We will exclude studies if they represent subgroup (e.g. women only) or post-hoc analyses of the original study. We will exclude *in vitro / in vivo* studies, animal studies, prospective cohort studies, retrospective cohort studies, case-control studies, cross-sectional studies, case reports / case series, conference abstracts / proceedings, narrative reviews, book abstracts, editorials / authors’ replies, and systematic reviews / meta-analyses (after scanning references for relevant articles).

*Types of Participants*: Studies evaluating human patients being managed for the prevention or treatment of CVA (ischemic or hemorrhagic stroke) of any age or sex. We will exclude animal studies.

*Types of Interventions / Exposures*: Studies were included if they evaluated management, drugs, procedures or devices for the treatment of CVA (ischemic or hemorrhagic stroke)

*Types of outcomes*:

Proportion of studies that excluded patients with renal disease

Study characteristics that are associated with exclusion of patients with renal disease

Proportion of studies that reported the proportion of enrolled patients with renal disease

Proportion of studies that reported baseline renal function

Proportion of studies that performed at least one subgroup analyses of any type

Proportion of studies that performed at least one subgroup analyses stratified by renal disease or function

- In short, we will include randomized controlled trials of management, drugs, procedures or devices for the management of patients with CVA (ischemic or hemorrhagic stroke), which had 100 or more participants and which report mortality outcomes.

**Data Sources**

*Search strategies*

We will perform a systematic search of MEDLINE database through the PUBMED interface from database inception through 07/01/2015. The search strategy will include articles indexed under both the Medical Subject Headings (Mesh) “*intracranial hemorrhage*” or “*stroke*” and also the Publication Type “Randomized Controlled Trial” (Table 2). The search will be limited to studies of human patients of any age or sex without language restrictions. We will restrict the search to medical journals that were listed as among the highest ten by annual impact factor journals in the fields of general internal medicine, neurology, cardiology, and nephrology in at least one year from 1999 to 2014. These included 23 general internal medicine journals, 28 neurology journals, 33 cardiology journals and 21 nephrology journals (Table 3-6. We will supplement this search by reviewing additional references from identified articles, not limited to the 105 major medical journals.

*Selection of studies*

Two review authors will independently screen all titles and abstracts obtained to identify potentially relevant trials. Two review authors will independently review the full text of the reports of these trials against the inclusion criteria. They will assess the eligibility of the trials independently and any differences in opinion will be settled by consensus. If necessary, a third review author will resolve disagreements. Trials will be included if they were randomized controlled trials of management of cerebrovascular accident (ischemic or hemorrhagic stroke) and had 100 or more participants randomized. Trials will be excluded if they did not report mortality outcomes (either as an endpoint or in safety/adverse event analysis) or were subgroup (e.g. elderly only), follow-up, or post-hoc analyses of the original study. The kappa statistic for inter-reviewer agreement will be calculated at each step.

**Data Extraction**

Two review authors will independently extract relevant study characteristics in duplicate using a standardized and piloted data extraction form. Disagreements will be resolved by consensus and, if necessary, a third review author. Variables will include: journal name, year of publication, study recruitment period, number of centers involved (single- vs multicenter), location (by continent of the first author; Europe, United States/Canada, or Asia/Australia/South America), number of patients randomized in each arm, funding source (academic or government grant, industry, both, or not specified), diagnostic category (ischemic vs. hemorrhagic stroke), treatment intervention implemented for each randomization arm, treatment class, whether these treatment interventions are listed as either class I or class II recommendations in the current AHA/ASA guidelines for the management of CVA, whether the protocol was registered in a clinical trials registry, number of patients randomized, whether patients with kidney disease were excluded, threshold of exclusion of kidney disease patients based on laboratory measurements such as serum creatinine, creatinine clearance, or estimated glomerular filtration rate (eGFR), renal replacement therapy, or nonspecific qualitative term, whether presence of kidney disease was an inclusion criterion, reported indices of baseline renal function for each randomization arm (i.e. mean or median creatinine, creatinine clearance or eGFR), reported proportion of participants with kidney disease in each arm, number of subgroup analyses by any non-renal baseline characteristics, and number of subgroup analyses by renal parameters. For studies with multiple reports, the first published report of trial results will be used. In the case of insufficient information in the index report, methods reports (without restriction to journal of publication) and registered protocols of the selected trials were reviewed to supplement and complete the data fields.

**Data Synthesis**

We will evaluate the variation of exclusion and reporting of renal disease by trial and article characteristics. The Fisher exact test will be used to evaluate differences in exclusion for renal disease among categorical variables. All variables with significant *P* values on univariate analysis will be included in the multivariate logistic regression analysis to identify independent predictors of exclusion of renal disease. Referent groups for each variable will be chosen by clinical judgment or by the group with the lowest frequency of exclusion. All analyses will be performed with STATA Version 12, College Station, TX. *P* values of <0.05 will considered statistically significant.

Table 1. Inclusion / Exclusion criteria

| Aspect | Inclusion Criteria |  | Exclusion Criteria |
| --- | --- | --- | --- |
| *Language / Date* | All languages / From inception through 07/18/2015 |  |  |
|  |  |  |  |
| *Publication status* | Published |  | Unpublished |
|  |  |  |  |
| *Design* | Randomized controlled trials |  | Observational studies (prospective cohort / retrospective cohort / case-control / cross-sectional) |
|  |  |  | *In vitro/ in vivo studies* |
|  |  |  | Systematic reviews / Meta-analyses* |
|  |  |  | Conferences' abstracts / proceedings** |
|  |  |  | Uncontrolled trials |
|  |  |  | Narrative reviews |
|  |  |  | Book abstracts |
|  |  |  | Editorials / Authors' reply |
|  |  |  |  |
| *Participants* | Studies on human patients of any age or sex |  | Animal studies |
|  |  |  |  |
| *Intervention / Exposure* | Management, drugs, procedures or devices for the treatment of CVA (ischemic or hemorrhagic stroke) |  |  |
|  |  |  |  |
| *Outcomes* | Studies that reported mortality outcomes (either as an endpoint or in safety/adverse event analysis) |  |  |

Table 2. Search strategy for selected journals through MEDLINE

|  | Query | Hits |
| --- | --- | --- |
| 1 | "Intracranial Hemorrhage"[Mesh]” | 57483 |
| 2 | "Stroke"[Mesh] | 91523 |
| 3 | 1 OR 2 | 142737 |
| 4 | "Randomized Controlled Trial" [Publication Type] | 391410 |
| 5 | 3 AND 4 | 5067 |
| 6 | 5 Filter: Humans | 5045 |
| 7 | 6 Filter: Publication date from inception to 07/18/2015 | 5045 |
| 8 | Selected journals only | 1558 |

**Table 3. Top ten journals for general internal medicine by impact from 2006 to 2014 based on Journal Citation Reports (ISI Web Of Knowledge)**

| **Year** | **First** | **Second** | **Third** | **Fourth** | **Fifth** | **Sixth** | **Seventh** | **Eighth** | **Ninth** | **Tenth** |
| --- | --- | --- | --- | --- | --- | --- | --- | --- | --- | --- |
| 2014 | NEJM | LANCET | JAMA | ANN INTERN MED | BRIT MED J | ARCH INTERN MED | PLOS MED | JAMA INTERN MED | J CACHEXIA SARCOPENI | BMC MED |
| 2013 | NEJM | LANCET | JAMA | BRIT MED J | ANN INTERN MED | PLOS MED | ARCH INTERN MED | J CACHEXIA SARCOPENI | BMC MED | COCHRANE DB SYST REV |
| 2012 | NEJM | LANCET | JAMA | BRIT MED J | PLOS MED | ANN INTERN MED | ARCH INTERN MED | BMC MED | CAN MED ASSOC J | J INTERN MED |
| 2011 | NEJM | LANCET | JAMA | ANN INTERN MED | PLOS MED | BRIT MED J | ARCH INTERN MED | CAN MED ASSOC J | BMC MED | COCHRANE DB SYST REV |
| 2010 | NEJM | LANCET | JAMA | ANN INTERN MED | PLOS MED | BRIT MED J | ANNU REV MED | ARCH INTERN MED | CAN MED ASSOC J | COCHRANE DB SYST REV |
| 2009 | NEJM | LANCET | JAMA | ANN INTERN MED | BRIT MED J | PLOS MED | ANNU REV MED | ARCH INTERN MED | CAN MED ASSOC J | J INTERN MED |
| 2008 | NEJM | JAMA | LANCET | ANN INTERN MED | BRIT MED J | PLOS MED | ANNU REV MED | ARCH INTERN MED | CAN MED ASSOC J | ANN MED |
| 2007 | NEJM | LANCET | JAMA | ANN INTERN MED | ANNU REV MED | PLOS MED | BRIT MED J | ARCH INTERN MED | CAN MED ASSOC J | ANN MED |
| 2006 | NEJM | LANCET | JAMA | ANN INTERN MED | PLOS MED | ANNU REV MED | BRIT MED J | ARCH INTERN MED | CAN MED ASSOC J | MEDICINE |
| 2005 | NEJM | LANCET | JAMA | ANN INTERN MED | ANNU REV MED | BRIT MED J | PLOS MED | ARCH INTERN MED | CAN MED ASSOC J | MEDICINE |
| 2004 | NEJM | JAMA | LANCET | ANN INTERN MED | ANNU REV MED | ARCH INTERN MED | BRIT MED J | CAN MED ASSOC J | AM J MED | MAYO CLIN PROC |
| 2003 | NEJM | JAMA | LANCET | ANN INTERN MED | ANNU REV MED | BRIT MED J | ARCH INTERN MED | CAN MED ASSOC J | MEDICINE | AM J MED |
| 2002 | NEJM | JAMA | LANCET | ANN INTERN MED | ANNU REV MED | BRIT MED J | ARCH INTERN MED | MEDICINE | AM J MED | ANN MED |
| 2001 | NEJM | JAMA | LANCET | ANN INTERN MED | ANNU REV MED | ARCH INTERN MED | BRIT MED J | AM J MED | MEDICINE | P ASSOC AM PHYSICIAN |
| 2000 | NEJM | JAMA | LANCET | ANNU REV MED | ANN INTERN MED | ARCH INTERN MED | AM J MED | BRIT MED J | MEDICINE | AMYLOID |
| 1999 | NEJM | JAMA | LANCET | ANN INTERN MED | ANNU REV MED | ARCH INTERN MED | BRIT MED J | MEDICINE | BRI MED BULL | J INVEST MED |

**Table 4. Top ten journals for nephrology by impact from 2006 to 2014 based on Journal Citation Reports (ISI Web Of Knowledge)**

| **Year** | **First** | **Second** | **Third** | **Fourth** | **Fifth** | **Sixth** | **Seventh** | **Eighth** | **Ninth** | **Tenth** |
| --- | --- | --- | --- | --- | --- | --- | --- | --- | --- | --- |
| 2014 | KIDINEY INT SUPP | J AM SOC NEPHROL | KIDNEY INT | NAT REV NEPHROL | AM J KIDNEY DIS | CLIN J AM SOC NEPHRO | CURR OPIN NEPHROL HY | NEPHROL DIAL TRANSPL | SEMIN NEPHROL | AM J PHYSIOL RENAL |
| 2013 | J AM SOC NEPHROL | KIDNEY INT | NAT REV NEPHROL | AM J KIDNEY DIS | CLIN J AM SOC NEPHRO | CURR OPIN NEPHROL HY | NEPHROL DIAL TRANSPL | AM J PHYSIOL RENAL | SEMIN NEPHROL | PEDIATR NEPHROL |
| 2012 | J AM SOC NEPHROL | NAT REV NEPHROL | KIDNEY INT | AM J KIDNEY DIS | CLIN J AM SOC NEPHRO | CURR OPIN NEPHROL HY | AM J PHYSIOL RENAL | NEPHROL DIAL TRANSPL | PEDIATR NEPHROL | SEMIN NEPHROL |
| 2011 | J AM SOC NEPHROL | NAT REV NEPHROL | KIDNEY INT | NAT CLIN PRACT NEPHR | AM J KIDNEY DIS | CLIN J AM SOC NEPHRO | CURR OPIN NEPHROL HY | AM J PHYSIOL RENAL | NEPHROL DIAL TRANSPL | NEPHRON PHYSIOL |
| 2010 | J AM SOC NEPHROL | KIDNEY INT | NAT CLIN PRACT NEPHR | AM J KIDNEY DIS | CLIN J AM SOC NEPHRO | NAT REV NEPHROL | CURR OPIN NEPHROL HY | AM J PHYSIOL RENAL | NEPHROL DIAL TRANSPL | NEPHRON EXP NEPHROL |
| 2009 | J AM SOC NEPHROL | KIDNEY INT | AM J KIDNEY DIS | NAT CLIN PRACT NEPHR | CLIN J AM SOC NEPHRO | AM J PHYSIOL RENAL | AM J NEPHROL | SEMIN DIALYSIS | NEPHROL DIAL TRANSPL | NEPHRON PHYSIOL |
| 2008 | J AM SOC NEPHROL | KIDNEY INT | NAT CLIN PRACT NEPHR | AM J KIDNEY DIS | CLIN J AM SOC NEPHRO | CURR OPIN NEPHROL HYP | AM J PHYSIOL RENAL | NEPHROL DIAL TRANSPL | AM J NEPHROL | SEMIN NEPHROL |
| 2007 | J AM SOC NEPHROL | KIDNEY INT | NAT CLIN PRACT NEPHR | AM J PHYSIOL RENAL | CURR OPIN NEPHROL HYP | AM J KIDNEY DIS | NEPHROL DIAL TRANSPL | SEMIN DIALYSIS | CLIN J AM SOC NEPHRO | AM J NEPHROL |
| 2006 | J AM SOC NEPHROL | KIDNEY INT | AM J PHYSIOL RENAL | CURR OPIN NEPHROL HYP | AM J KIDNEY DIS | NEPHROL DIAL TRANSPL | SEMIN NEPHROL | SEMIN DIALYSIS | AM J NEPHROL | PERITON DIALYSIS INT |
| 2005 | J AM SOC NEPHROL | KIDNEY INT | AM J KIDNEY DIS | AM J PHYSIOL RENAL | CURR OPIN NEPHROL HYP | NEPHROL DIAL TRANSPL | SEMIN NEPHROL | SEMIN DIALYSIS | AM J NEPHROL | KIDNEY BLOOD PRESSURE R |
| 2004 | J AM SOC NEPHROL | KIDNEY INT | AM J PHYSIOL RENAL | AM J KIDNEY DIS | CURR OPIN NEPHROL HYP | NEPHROL DIAL TRANSPL | SEMIN DIALYSIS | SEMIN NEPHROL | EXP NEPHROL | NEPHRON |
| 2003 | J AM SOC NEPHROL | KIDNEY INT | AM J PHYSIOL RENAL | CURR OPIN NEPHROL HYP | AM J KIDNEY DIS | NEPHROL DIAL TRANSPL | SEMIN NEPHROL | SEMIN DIALYSIS | PERITON DIALYSIS INT | EXP NEPHROL |
| 2002 | J AM SOC NEPHROL | AM J PHYSIOL RENAL | KIDNEY INT | AM J KIDNEY DIS | CURR OPIN NEPHROL HYP | NEPHROL DIAL TRANSPL | SEMIN NEPHROL | SEMIN DIALYSIS | PERITON DIALYSIS INT | EXP NEPHROL |
| 2001 | J AM SOC NEPHROL | KIDNEY INT | AM J PHYSIOL RENAL | AM J KIDNEY DIS | CURR OPIN NEPHROL HYP | NEPHROL DIAL TRANSPL | SEMIN NEPHROL | KIDNEY BLOOD PRESSURE R | EXP NEPHROL | NEPHRON |
| 2000 | J AM SOC NEPHROL | KIDNEY INT | AM J PHYSIOL RENAL | AM J KIDNEY DIS | CURR OPIN NEPHROL HYP | SEMIN NEPHROL | NEPHROL DIAL TRANSPL | PERITON DIALYSIS INT | NEPHRON | EXP NEPHROL |
| 1999 | J AM SOC NEPHROL | KIDNEY INT | AM J PHYSIOL RENAL | AM J KIDNEY DIS | SEMIN NEPHROL | PERITON DIALYSIS INT | CURR OPIN NEPHROL HYP | NEPHROL DIAL TRANSPL | NEPHRON | CLIN NEPHROL |

**Table 5. Top ten journals for neurology by impact from 2006 to 2014 based on Journal Citation Reports (ISI Web Of Knowledge)**

| **Year** | **First** | **Second** | **Third** | **Fourth** | **Fifth** | **Sixth** | **Seventh** | **Eighth** | **Ninth** | **Tenth** |
| --- | --- | --- | --- | --- | --- | --- | --- | --- | --- | --- |
| 2014 | LANCET NEUROL | NAT REV NEUROL | ALZHEIMERS DEMENT | ACTA NEUROPATHOL | ANN NEUROL | BRAIN | SLEEP MED REV | NEUROLOGY | ARCH NEUROL-CHICAGO | JAMA NEUROL |
| 2013 | LANCET NEUROL | ALZHEIMERS DEMENT | NAT REV NEUROL | ANN NEUROL | BRAIN | ACTA NEUROPATHOL | SLEEP MED REV | NEUROLOGY | NEUROSCIENTIST | ARCH NEUROL-CHICAGO |
| 2012 | LANCET NEUROL | NAT REV NEUROL | ALZHEIMERS DEMENT | ANN NEUROL | BRAIN | ACTA NEUROPATHOL | SLEEP MED REV | NEUROLOGY | ARCH NEUROL-CHICAGO | NEURO-ONCOLOGY |
| 2011 | LANCET NEUROL | NAT REV NEUROL | ANN NEUROL | PAIN PHYSICIAN | BRAIN | ACTA NEUROPATHOL | NEUROLOGY | NAT CLIN PRACT NEURO | ARCH NEUROL-CHICAGO | SLEEP MED REV |
| 2010 | LANCET NEUROL | ANN NEUROL | BRAIN | NEUROLOGY | PAIN PHYSICIAN | NAT CLIN PRACT NEURO | ACTA NEUROPATHOL | ARCH NEUROL-CHICAGO | NAT REV NEUROL | SLEEP MED REV |
| 2009 | LANCET NEUROL | BRAIN | ANN NEUROL | NEUROLOGY | STROKE | ACTA NEUROPATHOL | NAT CLIN PRACT NEURO | ARCH NEUROL-CHICAGO | NEUROSCIENTIST | SLEEP MED REV |
| 2008 | LANCET NEUROL | ANN NEUROL | BRAIN | NEUROLOGY | NAT CLIN PRACT NEURO | STROKE | SLEEP MED REV | PAIN | NEUROSCIENTIST | ARCH NEUROL-CHICAGO |
| 2007 | LANCET NEUROL | ANN NEUROL | BRAIN | STROKE | NEUROLOGY | NEURO-ONCOLOGY | NEUROSCIENTIST | ARCH NEUROL-CHICAGO | SLEEP MED REV | CURR OPIN NEUROL |
| 2006 | LANCET NEUROL | ANN NEUROL | BRAIN | CEPHALALGIA | NEUROSCIENTIST | NEUROLOGY | STROKE | BRAIN PATHOL | CURR OPIN NEUROL | ARCH NEUROL-CHICAGO |
| 2005 | LANCET NEUROL | ANN NEUROL | BRAIN | NEUROSCI BIOBEHAV R | STROKE | NEUROLOGY | SLEEP | ARCH NEUROL-CHICAGO | CURR OPIN NEUROL | BIPOLAR DISORD |
| 2004 | LANCET NEUROL | BRAIN | ANN NEUROL | NEUROSCI BIOBEHAV R | NEUROLOGY | STROKE | J NEUROPATH EXP NEUR | SLEEP | ARCH NEUROL-CHICAGO | INT J NEUROPSYCHOPH |
| 2003 | BRAIN | ANN NEUROL | NEUROLOGY | NEUROSCI BIOBEHAV R | STROKE | J NEUROPATH EXP NEUR | ARCH NEUROL-CHICAGO | PAIN | INT J NEUROPSYCHOPH | CURR OPIN NEUROL |
| 2002 | ANN NEUROL | BRAIN | BRAIN PATHOL | NEUROSCI BIOBEHAV R | NEUROLOGY | STROKE | J NEUROPATH EXP NEUR | PAIN | ARCH NEUROL-CHICAGO | CURR OPIN NEUROL |
| 2001 | BRAIN PATHOL | ANN NEUROL | BRAIN | J NEUROPATH EXP NEUR | STROKE | NEUROLOGY | NEUROSCI BIOBEHAV R | PAIN | SLEEP | ARCH NEUROL-CHICAGO |
| 2000 | ANN NEUROL | BRAIN | BRAIN PATHOL | SCHIZOPHRENIA BULL | STROKE | J NEUROPATH EXP NEUR | J CLIN PSYCHOPHARM | NEUROLOGY | ARCH NEUROL-CHICAGO | PAIN FORUM |
| 1999 | ANN NEUROL | BRAIN | SCHIZOPHRENIA BULL | J CLIN PSYCHOPHARM | J NEUROPATH EXP NEUR | STROKE | NEUROLOGY | NEUROBIOL DIS | BRAIN PATHOL | PAIN |

**Table 6. Top ten journals for cardiology by impact from 2006 to 2014 based on Journal Citation Reports (ISI Web Of Knowledge)**

| **Year** | **First** | **Second** | **Third** | **Fourth** | **Fifth** | **Sixth** | **Seventh** | **Eighth** | **Ninth** | **Tenth** |
| --- | --- | --- | --- | --- | --- | --- | --- | --- | --- | --- |
|  | J AM COLL CARDIOL | EUR HEART J SUPPL | EUR HEART J | CIRCULATION | CIRC RES | NAT REV CARDIOL | JACC – CARDIOVASC INTERV | JACC – CARDIOVASC IMAG | EUR J HEART FAIL | CIRC – CARDIOVASC INTERV |
| 2013 | J AM COLL CARDIOL | CIRCULATION | EUR HEART J | CIRC RES | NAT REV CARDIOL | JACC – CARDIOVASC INTERV | JACC – CARDIOVASC IMAG | CIRC – CARDIOVASC INTERV | CIRC – CARDIOVASC IMAG | EUR J HEART FAIL |
| 2012 | CIRCULATION | EUR HEART J | J AM COLL CARDIOL | CIRC RES | NAT REV CARDIOL | CIRC-CARDIOVASC GENE | CIRC-HEART FAIL | JACC – CARDIOVASC INTERV | CIRC – CARDIOVASC INTERV | JACC – CARDIOVASC IMAG |
| 2011 | CIRCULATION | J AM COLL CARDIOL | EUR HEART J | CIRC RES | NAT REV CARDIOL | BASIC RES CARDIOL | INT J CARDIOL | NAT CLIN PRACT CARD | JACC – CARDIOVASC INTERV | CIRC - ARRHYTHMIAELEC |
| 2010 | CIRCULATION | J AM COLL CARDIOL | EUR HEART J | CIRC RES | NAT REV CARDIOL | INT J CARDIOL | NAT CLIN PRACT CARD | CARDIOVASC RES | JACC – CARDIOVASC INTERV | JACC – CARDIOVASC IMAG |
| 2009 | CIRCULATION | J AM COLL CARDIOL | EUR HEART J | CIRC RES | BASIC RES CARDIOL | NAT CLIN PRACT CARD | HEART FAIL REV | CARDIOVASC RES | HEART | CARDIOVASC DRUG REV |
| 2008 | CIRCULATION | J AM COLL CARDIOL | CIRC RES | EUR HEART J | NAT CLIN PRACT CARD | CARDIOVASC RES | BASIC RES CARDIOL | J MOL CELL CARDIOL | HEART | AM HEART J |
| 2007 | CIRCULATION | J AM COLL CARDIOL | CIRC RES | EUR HEART J | CARDIOVASC RES | J MOL CELL CARDIOL | TRENDS CARDIOVASCMED | NAT CLIN PRACT CARD | BASIC RES CARDIOL | HEART RHYTHM |
| 2006 | CIRCULATION | CIRC RES | J AM COLL CARDIOL | EUR HEART J | CARDIOVASC RES | J MOL CELL CARDIOL | TRENDS CARDIOVASCMED | BASIC RES CARDIOL | HEART RHYTHM | AM J PHYSIOL – HEART C |
| 2005 | CIRCULATION | CIRC RES | J AM COLL CARDIOL | EUR HEART J | CARDIOVASC RES | J MOL CELL CARDIOL | HEART | CURR PROB CARDIOLOGY | AM J PHYSIOL – HEART C | AM HEART J |
| 2004 | CIRCULATION | CIRC RES | J AM COLL CARDIOL | EUR HEART J | TRENDS CARDIOVAS MED | CARDIOVASC RES | J MOL CELL CARDIOL | AM HEART J | AM J PHYSIOL – HEART C | HEART |
| 2003 | CIRCULATION | CIRC RES | J AM COLL CARDIOL | EUR HEART J | CARDIOVASC RES | J MOL CELL CARDIOL | TRENDS CARDIOVAS MED | AM J PHYSIOL – HEART C | J THORAC CARDIOV SUR | AM HEART J |
| 2002 | CIRCULATION | CIRC RES | J AM COLL CARDIOL | EUR HEART J | CARDIOVASC RES | J MOL CELL CARDIOL | TRENDS CARDIOVAS MED | AM J PHYSIOL – HEART C | J CARDIOVASC ELECTR | CHEST |
| 2001 | CIRCULATION | CIRC RES | J AM COLL CARDIOL | EUR HEART J | CARDIOVASC RES | J MOL CELL CARDIOL | TRENDS CARDIOVAS MED | AM J PHYSIOL – HEART C | J CARD FAIL | AM HEART J |
| 2000 | CIRCULATION | CIRC RES | J AM COLL CARDIOL | EUR HEART J | CARDIOVASC RES | J MOL CELL CARDIOL | AM J PHYSIOL – HEART C | TRENDS CARDIOVAS MED | J CARDIOVASC ELECTR | AM J CARDIOL |
| 1999 | CIRCULATION | CIRC RES | J AM COLL CARDIOL | PROG CARDIOVASC DIS | EUR HEART J | CARDIOVASC RES | J MOL CELL CARDIOL | AM J PHYSIOL – HEART C | CHEST | AM J CARDIOL |
